# Supplementary material for: WordCommentsAnalyzer: A windows software tool for qualitative research
Source: F1000Res. 2018 Sep 4;7:536. Originally published 2018 May 3. [Version 2] doi: 10.12688/f1000research.14819.2 (PMC6137411; doi:10.12688/f1000research.14819.2)
Supplement: Supplementary file 3 [file f1000research-7-17614-s0002.tgz › b2a79496-4ada-47c4-9682-a99ce867836d.zip!/Eight Iranian Health Journals/Archives of Rehabilitation_66.docx]

© Archives of Rehabilitation. This work is licensed under CC BY-NC 4.0 (https://creativecommons.org/licenses/by-nc/4.0/).

Volume & Issue: دوره 3 - شماره 4

Title: بررسی مقایسه ای کارکردهای غلبه طرفی مغز در کودکان عقب مانده ذهنی با سن عقلی ۶–۵ ساله مراکز آموزش و پرورش استثنایی با کودکان عادی

http://rehabilitationj.uswr.ac.ir/article-1-576-fa.html

هدف: بررسی رشد روانی–حرکتی کودکان از جمله مقوله های مورد توجه متخصصان اطفال و توان بخشی می باشد. این رشد وابسته به چگونگی روند تکامل سیستم اعصاب مرکزی می باشد و لذا هرگونه اختلال در روند تکامل این سیستم می تواند باعث بروز اختلالات قابل توجه در زمینه های حسی، حرکتی و شناختی شود. گذر طبیعی از مراحل مختلف رشدی پایه و اساس پیشرفت کودک برای نیل به سطح شناختی مطلوب می باشد. کودکان عقب مانده ی ذهنی علاوه بر نقص در توان مندی های ذهنی، در زمینه ی حرکات درشت، حرکات ظریف، مهارت های درکی–حرکتی، جهت یابی و تعادل نیز دارای اختلالات قابل توجه می باشند. صرف نظر از اتیولوژی بروز عقب ماندگی های ذهنی و اختلالات ناشی از آن، چگونگی تکامل کارکردی مغز در روند پردازش درون داده های حسی و برون داده های حرکتی، بستر مهمی را در توان بخشی این کودکان فراهم می آورد که باید مورد توجه قرار گیرد. در این میان توجه به پدیده های طرفی شدن کارکردی مغز در روند پردازش درون حسی و برون داده های حرکتی، بستر مهمی را در توان بخشی این کودکان فراهم می آورد که باید مورد توجه قرار گیرد. در این میان توجه به پدیده ی طرفی شدن کارکردی نیمکره های مغز، غلبه طرفی، که بخشی از روند تکامل طبیعی مغز می باشد، که در به کارگیری روش های درمانی حسی–حرکتی توان بخشی در مورد کودکان عقب مانده، بسیار مهم می باشد. از آنجا که تاکنون در زمینه بررسی مقایسه ای کارکردهای غلبه طرفی مغز در بین کودکان سالم و عقب مانده در کشور ما مطالعه ای انجام نشده است، روش بررسی: در تحقیق حاضر به مقایسه ی پارامترهای کارکردی معرف طرفی شدن مغز شامل غلبه ی طرفی بینایی، شنوایی، حرکات دست و حرکات پا در دو گروه کودکان سالم و عقب مانده با سن عقلی یکسان از طریق به کاربردن جدول ارزیابی رشد عصبی–مغزی دلاکاتو پرداختیم یافته‌ها: بررسی آماری نتایج به دست آمده نشان دهنده ی تفاوت معنادار در اغلب پارامترهای شاخص غلبه ی کارکردی طرفی مغز بین دو گروه کودکان یاد شده است. نتیجه‌گیری: با توجه به نتایج حاصل، اعتقاد ما بر این است که روند متفاوت طرفی شدن کارکردی مغز در کودکان عقب مانده ذهنی، دقت و احتمالا تجدید نظر در روش های توان بخشی این کودکان را ایجاب می نماید.

Title: بررسی ارتباط لکنت و برخی بیماریهای آلرژیک در شهر اصفهان، سال ۸۱-۱۳۸۰

http://rehabilitationj.uswr.ac.ir/article-1-577-fa.html

هدف: یک از رایج ترین و کامل ترین شیوه های برقراری ارتباط در انسان ها از طریق گفتار شکل می گیرد. گفتار هم در مراحل یادگیری و هم در مراحل اجرا داری پیچیدگی های فراوانی است و به همین دلیل در معرض اختلالات بسیاری قرار دارد. یکی از این اختلالات، لکنت است. لکنت نشانگانی است که فرد مبتلا، به دلیل از هم گسیختگی های غیر ارادی در جریان گفتار که غالبا توسط گیر، قفل، تکرار، کشیدگی بیش ازحد، مکث و ... نمایان می گردد، قادر نیست به طور طبیعی صحبت کند. عوامل متعددی در شکل گیری و تشدید لکنت موثرند. یکی از این عوامل که در منابع متعددی به آن اشاره شده، آلرژی ها هستند. حدود %15 از مردم جهان به نوعی از آلرژی مبتلا می باشند و از آن رنج می برند. در این پژوهش هدف، بررسی ارتباط میان لکنت و انواعی از آلرژی ها شامل آسم آلرژیک، رینیت و درماتیت می باشد. روش بررسی: در این پژوهش 33 بیمار لکنتی مراجعه کننده به کلینیک های گفتار درمانی به عنوان گروه مورد و 57 نفر از افراد غیر لکنتی همسان شده به عنوان گروه شاهد در نظر گرفته شدند. پرسش نامه ی مورد نظر برای بررسی آلرژی توسط تمام نمونه ها تکمیل و مورد بررسی قرار گرفت. یافته ها: در گروه مورد، از بین 33نفر لکنتی، 9نفر (%27/3) مبتلا به آسم آلرژیک و در گروه شاهد، از بین 57 نفر غیر لکنتی، 6 نفر (%10/5) مبتلا به آسم بوده اند. در مورد رینیت، در گروه مورد، از 33نفر لکنتی 6 نفر (%18/2) علائم رینیت را نشان داده و در گروه شاهد، 6نفر (%10/5) مبتلا به رینیت بوده اند. و در بین لکنتی های مزبور، 1نفر (%3) دارای درماتیت و ازغیر لکنتی ها نیز 4 نفر (%7) درماتیت داشته اند.  نتیجه گیری: بر اساس نتایج به دست آمده از این پژوهش بین لکنت و آسم آلرژیک بیماران لکنتی رابطه معنی دار به دست آمد ولی هیچ رابطه ی معنی داری میان دو نوع دیگر آلرژی مورد بررسی یعنی رینیت و درماتیت و لکنت حاصل نشد.

Title: بررسی شیوع اختلالات گفتار و زبان، بین دانش آموزان مقطع ابتدایی دخترانه

http://rehabilitationj.uswr.ac.ir/article-1-579-fa.html

هدف: این پژوهش با هدف بررسی شیوع اختلالات گفتار و زبان در بین دانش آموزان مقطع ابتدایی دخترانه در مناطق 3 و 19 آموزش و پرورش تهران در سال تحصیلی 1379-80 انجام شده است. روش بررسی: جامعه، مورد مطالعه، دانش آموزان دو منطقه ی 3 و 19 بودند که از میان آن ها 1010 دانش آموزش به طور تصادفی از میان کل دانش آموزان انتخاب گردیدند. (به طور متوسط از هر پایه 200 نفر) سپس پرسش نامه تکمیل می گردید و بعد گفتار دانش آموزش مورد ارزیابی قرار می‌گرفت تا در صورت وجود هر نوع اختلال در فرم مخصوص ضبط گردد، ابزار ارزیابی متن کتاب فارسی سال قبل دانش آموز و سری تصاویر به هم مربوط بود، هم چنین حفره ی دهان تمام دانش آموزان، از لحاظ حرکت وضع آناتومی اندام ها، و آکلوژن دندانی مورد ارزیابی قرار می گرفت. یافته ها: با توجه به نتایج حاصل از این تحقیق مشخص گردید که از میان 1010 دانش آموز، 163 نفر (%16/1) اختلال داشتند که از میان آن ها 75 دانش آموز (%46) مربوط به منطقه 19 و 88 دانش آموزش (%54) مربوط به منطقه 3 بودند. اختلالات مورد مطالعه در این تحقیق عبارت بودند از اختلال در تولید، لکنت، اختلال صوت، بلع معکوس، اختلال در تشدید، اختلال در خواندن.

Title: بررسی تأثیر آموزش راهبردهای فراشناختی بر عملکرد حل مسئله

http://rehabilitationj.uswr.ac.ir/article-1-580-fa.html

هدف:  هدف پژوهش حاضر تعیین نقش مهارت های فرا شناختی در عملکرد حل مساله، مقایسه ی شیوه های آموزش این مهارت ها و تعیین موثرترین شیوه برای آموزش مهارت های فراشناختی است  روش بررسی: به همین منظور 62 دانش آموز مقطع سوم راهنمایی شهر تهران به روش نمونه گیری خوشه ای چند مرحله ای انتخاب شدند. سپس تمامی آزمودنی گروه نمونه پرسش نامه ای به سبک اسناد کودکان و پرسش نامه ی فرا شناختی را تکمیل نمودند. هم چنین هر آزمودنی به طور انفرادی در معرض موقعیت آونگ قرار گرفت. سپس آزمودنی ها به طور تصادفی در سه گروه آزمایش آموزش ترکیبی، آموزش دو جانبه، آموزش اسناد و یک گروه کنترل قرار گرفتند و پس از انجام آموزش های فوق مجددا در معرض پس آزمون قرار داده شدند. یافته‌ها: نتایج حاصل از پیش آزمون و پس آزمون در گروه های فوق با استفاده از آزمون های ناپارامتریک مورد نجزیه و تحلیل قرار گرفت. یافته ها بیانگر این مطلب است که آموزش راهبردهای فرا شناختی بر عملکرد حل مساله دانش آموزان تاثیرات بسزایی دارد. نتیجه‌گیری: بررسی‌ها نشان داد گروه‌های آموزشی و گروه گواه از نظر عملکرد حل مسئله با یکدیگر تفاوت دارند که از نظر آماری معنادار است. به بیان دیگر اعضای شرکت‌کننده در هر یک از این گروه‌ها در مواجه با مسائل مطرح شده در پس‌آزمون نسبت به اعضای گروه گواه متفاوت عمل کردند. مقایسه روش‌های تدریس فراشناختی نشانگر این است که در کل آموزش ترکیبی به دلیل تصحیح سیستم انگیزش فرد از طرفی و آموزش کنترل و نظارت بر سیر دریافت حل مسئله و پیشنهاد راه حل با توجه به موقعیت مسئله تفاوت معناداری با هر یک از دوشیوه به تنهایی دارد.

Title: نقش توان‌بخشی در درمان سندرم خروجی قفسه سینه

http://rehabilitationj.uswr.ac.ir/article-1-583-fa.html

هدف: با بررسی مقالات و تاکید بر تاثیر فیزیوتراپی و توان بخشی عضلات شانه و گردن در درمان سندرم خروجی قفسه سینه، تصمیم گرفته شد تا بررسی دقیقی روی این بیماران صورت گیرد، لذا بیمارانی را که با تشخیص سندرم خروجی قفسه سینه به کلینیک مراجعه کردند تحت بررسی قرار گرفتند و به این نتیجه رسیدیم که فیزیوتراپی و توان بخشی عضلات شانه و گردن می تواند در بهبود بسیاری از بیماران موثر باشد.  روش بررسی: طی هفت سال، تعداد 131 مورد سندرم خروجی قفسه سینه تحت پی گیری و درمان و تعداد بیس و شش مورد (%20) مورد عمل جراحی قرار گرفتند. 84 مورد (%64) با درمان های کنسرواتیو بهبودی پیدا کردند، 21مورد (%16 ) راضی به عمل جراحی نشده و با ادامه درمان کنسرواتیو بهبودی نسبی پیدا کردند. بیماران برای مدت پنج سال تحت نظر بودند و برای نتبجه گیری رضایت آنها نسبت به از بین رفتن درد مورد توجه قرار گرفت. یافته ها: تمام بیماران در ابتدا برای مدت 4 ماه تحت درمان توانبخشی عضلانی و فیزیوتراپی قرار گرفتند و افرادی که در طی این مدت، بهتر نشدند، آمادگی خود را جهت عمل جراحی اعلام کردند. تعداد 26 بیمار که تحت عمل جراحی دنده اول قرار گرفتند، به دو گروه تقسیم کردیم، گروه اول 14 نفر که با روش فوق کلاویکول و گروه دوم 12 نفر که با روش زیر بغلی تحت درمان جراحی قرار گرفتند. روش فوق کلاویکول از نظر زمان، خونریزی، عارضه، و تعداد افراد کمک دهنده در طور عمل جراحی بهتر بود. نتیجه گیری: در حد امکان باید سعی شود؛ بیماران با روش توان بخشی عضلات شانه، گردن و تغییرات در وضعیت بیمار در هنگام نشستن، خوابیدن و کارکردن، مورد معالجه قرار گیرند و اگر بعد از چهارماه بهبودی حاصل نشد، جراحی برای بیماران اندیکاسیون دارد. عمل جراحی در مورد 23 نفر نتیجه خوبی داشت، دو نفر دیگر با عود بیماری روبه رو شدند که با فیزیوتراپی بهتر شد و دریک مورد نیز، نوروپارکسی دیده شد که بعد از 12ماه بهبودی حاصل گردید.

Title: بررسی اثرات روزه‌داری در میزان نزدیک‌بینی

http://rehabilitationj.uswr.ac.ir/article-1-584-fa.html

هدف: با توجه به امر عبادی روزه ماه مبارک رمضان، بررسی شایعات مخدوش و مبهمی که در اذهان عمومی مبنی بر تاثیر سوء آن در شماره چشم، کم و بیش وجود دارد، است. روش بررسی: پژوهش حاضر، بر روی 180 چشم (90بیمار) در دو گروه سنی 12-19 سال و 20-30 سال که همگی درجاتی از میوپی (نزدیک بینی) داشتند و بطور تصادفی از بین مراجعان به کلینیک عمومی چشم انتخاب شده بودند، مورد معاینه و بررسی قرار گرفتند. معاینات در دو مرحله، یک بار از سه روز قبل از شروع ماه رمضان تا شب قبل از ماه مبارک، یک بار از روز بعد از عید فطر تا سه شب بعد آن، توسط یک معاینه کننده، یک ست معاینه و تابلوی E.Chart ثابت انجام شد. یافته‌ها: بررسی نتایج آماری، تاثیر خاصی را در گروه مورد مطالعه نشان نداد و می توان گفت که روزه داری، تاثیر قابل توجهی بر نزدیک بینی ندارد.

Title: آموزش یکپارچگی شنیداری

http://rehabilitationj.uswr.ac.ir/article-1-585-fa.html

آموزش یکپارچگی شنیداری نوعی آموزش تقویت شنوایی است که درمان برخی اختلالات ورودی حسی نظیر اتیسم، اختلال بیش فعالی نقص توجهی (ADHD)، ناتوانی در خواندن، بیش فعالی، ناتوانی های یادگیری (LDS)، نقایص زبانی، اختلالات رشد فراگیر (PDD)، اختلالات پردازش شنوایی مرکزی (CAPD)، اختلال نقص توجهی (ADD)، افسردگی و حساسیت بیش از حد به صدا، کاربرد دارد. این روش درمانی چند سالی است که در ایالات متحده رواج یافته و از زمان انتشار کتاب (1991) The Sound of Miracle توسط خانم Annabel Stehli مورد توجه بسیاری قرار گرفته است. در این کتاب Stehli، تجارب مربوط به قبل و پس از استفاده از AIT را برای دختر چهار ساله‌اش که مبتلا به اوتیسم تشخیص داده شده بود، شرح داده است.
